# Common Variants in the TBX5 Gene Associated with Atrial Fibrillation in a Chinese Han Population

**DOI:** 10.1371/journal.pone.0160467

**Published:** 2016-08-01

**Authors:** Rongfeng Zhang, Xiaochen Tian, Lianjun Gao, Huihua Li, Xiaomeng Yin, Yingxue Dong, Yanzong Yang, Yunlong Xia

**Affiliations:** First Affiliated Hospital of Dalian Medical University, Dalian, China; Huashan Hospital, Fudan University, CHINA

## Abstract

**Background:**

PR interval variations have recently been associated with an increased risk of long-term atrial fibrillation (AF), heart block and all-cause mortality. Genome-wide association studies have linked the PR interval with several common variants in the *TBX5* gene. Several variants in the *TBX5* gene, including rs7312625 and rs883079, have been associated with AF. The purpose of this study was to determine the association of single-nucleotide polymorphisms (SNPs) in the *TBX5* gene, rs7312625 and rs883079, with AF in Chinese Han patients.

**Methodology/Principal Findings:**

In this case-control association study, large cohorts of AF patients (n = 1132) and controls (n = 1206) were recruited from different hospitals. The genotyping was performed using a Rotor-Gene TM 6000 high-resolution melt system. Rs7312625, rs3825214 and rs883079 were analyzed. We found that SNP 3825214 was significantly associated with AF (*P*-obs = 0.002, odds ratio [OR] = 0.82), and lone AF (*P*-obs = 6.77x10^-5^, odds ratio [OR] = 0.71). SNP rs7312625 was significantly associated with lone AF (*P*-obs = 0.015, odds ratio [OR] = 1.27), although its association with AF was not significant. No significant association of SNP rs883079 with AF or lone AF was observed. Thus, we analyzed the interaction among these three loci. We demonstrated significant interaction among rs3825214, rs7312625 and rs883079. Four-locus risk alleles showed the highest odds ratio in combined rs3825214 and rs7312625 (P-obs<0.0001, odds ratio [OR] = 2.21). Six-locus risk alleles showed the highest odds ratio in combined rs3825214, rs7312625 and rs 883079(P-obs<0.0001, odds ratio [OR] = 2.35). Significance was established with the trend test (P<0.0001).

**Conclusions:**

For the first time, we report the strong association of SNP rs3825214 in the *TBX5* gene with AF and lone AF in a Chinese Han population. Rs7312625 was significantly associated with lone AF, and snp-snp interaction increased the risk of atrial fibrillation. Our data might provide new insights into understanding AF pathogenesis and designing novel genetic therapies for AF patients.

## Introduction

Atrial fibrillation (AF) is the most common arrhythmia, affecting 1–2% of the general population. As the aging population continues to increase, the number of AF patients likewise increases. In the United States, the estimated number of AF patients has reached approximately 2 million [1]. In China, the estimated number of AF patients has reached approximately 8 million [2]. Many genes have been implicated in AF. The TBX5 gene is a member of the T-box transcriptional factors and plays an essential role in cardiac development [3]. Mutations in the TBX5 gene have been associated with cardiac defects [4, 5]. A gain-of-function mutation in the TBX5 gene has been linked to atypical Holt-Oram syndrome and paroxysmal AF [4, 6]. Recent genome-wide association studies have demonstrated that several common variants of the *TBX5* gene, including rs7312625 and rs883079, have been identified and associated with PR interval duration or AF in various ethnic populations [7–10]. However, these TBX5 gene variants have not been independently studied in AF patients in a Chinese Han population.

The PR interval is the interval between the beginning of the P wave and the beginning of the QRS complex on an electrocardiogram, representing the intra- and inter-atrial conduction time. PR interval prolongation is an independent predictor for increased AF risk [10–12]. The PR interval, as an amalgamated measure of atrial and atrioventricular nodal conduction, has been independently associated with increased AF risk. Identification of the genetic variants underlying the heritability of the PR interval might provide novel insights into facilitating genetic prediction of the AF risk. In our previous studies, we found a strong association of the TBX5 variant rs3825214 with AF, providing supporting evidence for the results of genome-wide association studies [13].

In this study, we went further, assessing the association between the TBX5 gene variants rs7312625 and rs883079 with AF in a large group of Chinese Han patients. A case-control association study was conducted with 1132 AF patients and 1206 controls. We found that although rs7312625 was not associated with AF, it was significantly associated with lone atrial fibrillation (*P* = 0.015).

## Methods

### Study population

The research was approved by the ethics committee of the First Affiliated Hospital of Dalian Medical University. The data were analyzed anonymously. The AF samples from the patients were from the Chinese GeneID database. The Chinese GeneID database has been created for identification of the genes involved in cardiovascular diseases, including AF, coronary artery disease (CAD), stroke and essential hypertension (HT), in mainland China [13, 14]. AF was diagnosed according to the American College of Cardiology/American Heart Association/European Society of Cardiology (ACC/AHA/ESC) 2010 guidelines (Fuster et al. 2010). AF without HT, CAD, hyperthyroidism, cardiomyopathies, valvulopathies, or other cardiac diseases was classified as lone AF (Fuster et al. 2006). Patients with hyperthyroidism, cardiomyopathies and valvular heart disease were excluded from this study. The clinical data included age, gender, and a history of HT, diabetes, CAD, stroke and hyperthyroidism. HT was defined as blood pressure >140/90 mmHg or a history of medication for HT. Individuals with a coronary stenosis of 70%, percutaneous coronary angioplasty, coronary artery bypass graft, or myocardial infarction were classified as CAD. Diabetes mellitus was diagnosed by a fasting blood glucose level of C7 mmol/L. Echocardiography was preformed to exclude cardiomyopathies and valvular heart disease. Hyperthyroidism was investigated by physical examinations and thyroid function testing. The controls had no history of AF, hyperthyroidism, cardiomyopathies and valvular heart disease. To minimize the subpopulation effect, the AF patients were matched with controls from the same geographical areas.

### Genotyping

Blood samples were collected from the study participants, and the genomic DNA samples were purified using a Genomic DNA Purification Kit (Promega Corporation, Madison, WI, USA) according to the manufacturer's protocol. The genotyping for single-nucleotide polymorphisms (SNPs) was performed using Syto9 green fluorescent dye-based high-resolution melt analysis on a Rotor-gene 6200 System (Corbett Life Science, Concorde, NSW, Australia) according to the manufacturers’ protocols. Briefly, a DNA fragment flanking SNPs was amplified by PCR with a final 5 μM concentration of Syto9 green fluorescent dye. The dissociation behaviors of the two different alleles were assessed to determine the genotypes, as the double-stranded DNA was transited into single-stranded DNA with temperature increases. To ensure quality, DNA samples with previously known genotypes were used as the positive controls, and the appropriate negative controls were included. The genotypes were validated using direct sequencing in 50 randomly selected subjects.

### Statistical analysis

The SNPs were tested for Hardy-Weinberg equilibrium in the controls using PLINK v1.05. The haplotype construction and frequencies were estimated using Haploview v4.2 software. The allelic and genotypic associations of the SNPs with AF were assessed using Pearson’s 2×2 and 2×3 contingency table χ2 test (PLINK v1.05). The odds ratios (ORs) and 95% confidence intervals (CIs) were estimated using the χ2 test (PLINK v1.05). The multivariate analysis was performed by incorporating age, sex, HT and type 2 diabetes as the covariants using multivariate logistic regression (SPSS 17.0). The empirical P values were determined using the PLINK v1.05 program with 100,000 Monte-Carlo simulations. The comparisons of the clinical parameters were tested using Student’s *t*-test for the continuous variables, which are expressed as the means ± SD. The *χ*^2^ test was performed using SPSS 17.0 for the categorical variables. Correct *P* values were obtained using Bonferroni’s correction as *P*-cor = 1-(1-*P*-obs)^n^. The power analysis was performed using Power and Sample Size Program 3.0.

## Results

### Baseline characteristics of the study population

In total, 1132 AF patients and 1206 controls from the GeneID database were enrolled in this study. The baseline characteristics of the patients are shown in Table 1. The AF cohort included paroxysmal (n = 611, 53.98%), persistent (n = 430, 37.99%) and permanent (n = 91, 8.04%) AF patients. Less than half of the AF patients (n = 356, 31.45%) were diagnosed as lone AF. The frequency of HT in the AF patients was significantly higher than in the controls (*P* = 2.39e-17). The frequencies of type 2 diabetes in the AF patients and controls were similar (*P* = 0.15).

**Table 1:** Clinical Characteristics of the Study Populations.

	AF patients(n = 1132)	Controls(n = 1206)	*P* value
Age (years)*	65.5±12.9	59.8±11.3	6.43e-13
Sex, female n (%)	486 (42.93)	490 (40.63)	0.30
Hypertension^†^ n (%)	624 (55.12)	454 (37.65)	2.39e-17
Diabetes n (%)^‡^	121 (10.69)	152 (12.60)	0.15
AF category			
Paroxysmal n (%)	611 (53.98)	n.a.	n.a.
Persistent n (%)	430 (37.99)	n.a.	n.a.
Permanent n (%)	91 (8.04)	n.a.	n.a.
Long AF n (%)	356 (31.45)	n.a.	n.a.

n.a.: not applicable.

* Age for the AF patient group refers to the age at the diagnosis of AF; age for the control group refers to the age at which the study subjects were enrolled into the study.

^†^ Hypertension was diagnosed by the criteria of blood pressure higher than 140/90 mmHg.

^‡^ Diabetes was defined as ongoing treatment of diabetes or a fasting plasma glucose level of ≥ 7.0 mmol/L.

### The SNPs in *TBX5* and AF

There was a significant association between the G allele in rs3825214 and AF (*P* = 0.036; *P*_*adj*_ = 0.025, OR = 0.790). In both the all AF and lone AF groups, the distributions of rs3825214 were significantly different than those of the control group (*P* = 0.029 and 0.003, respectively).

In the controls, there was no deviation from Hardy-Weinberg equilibrium for the SNPs. The distributions of the allele from the analysis are summarized in Table 2. SNP rs7312625 was not associated with AF in either the entire cohort or the male and female groups. SNP rs7312625 was significantly associated with the lone AF group (*P*-obs = 0.015, OR = 1.27). In addition, there was no significant association between SNP rs883079 and AF in either the entire cohort or the male and female groups.

**Table 2:** Association of the SNPs in *TBX5* with AF.

	Minor allele frequency	Without adjustment*		With adjustment^†^		Empirical *P*^‡^
	Case/Control	*P-obs*	OR (95% CI)	*P-adj*	OR (95% CI)	*P-emp*
Rs3825214						
Entire cohort	0.443/0.492	0.002	0.82 (0.73–0.93)	0.001	1.23 (1.09–1.39)	0.001
Male	0.430/0.484	0.006	0.80 (0.69–0.94)	0.008	0.82 (0.71–0.95)	0.005
Female	0.462/0.503	0.09	0.85 (0.70–1.02)	0.12	0.88 (0.74–1.06)	0.09
AF with HT	0.447/0.504	0.01	0.80 (0.67–0.95)	0.02	0.83 (0.7100.98)	0.02
AF without HT	0.440/0.484	0.03	0.84 (0.71–0.99)	0.06	0.85 (0.72–1.01)	0.03
Lone AF	0.406/0.492	6.77x10^-5^	0.71 (0.60–0.84)	3.85x10^-5^	0.77 (0.66–0.89)	8.34x10^-5^
Other AF	0.470/0.492	0.18	0.90 (0.75–1.06)	0.15	0.88 (0.74–1.07)	0.16
Rs7312625						
Entire cohort	0.257/0.247	0.43	1.06 (0.92–1.21)	0.40	1.05 (0.91–1.19)	0.28
Male	0.268/0.252	0.35	1.09 (0.91–1.31)	0.38	1.07 (0.90–1.28)	0.20
Female	0.242/0.241	0.94	1.00 (0.81–1.26)	0.96	1.01 (0.82–1.26)	1
AF with HT	0.263/0.238	0.22	1.14 (0.92–1.41)	0.26	1.18 (0.96–1.48)	0.18
AF without HT	0.250/0.252	0.90	0.99 (0.81–1.20)	0.94	0.95 (0.79–1.17)	0.67
Lone AF	0.294/0.247	0.015	1.27 (1.05–1.54)	0.011	1.29 (1.08–1.55)	0.01
Other AF	0.232/0.247	0.55	0.92 (0.88–1.24)	0.68	0.96 (0.90–1.27)	0.50
Rs883079						
Entire cohort	0.439/0.424	0.33	1.06 (0.94–1.21)	0.28	1.08 (0.96–1.25)	0.27
Male	0.442/0.408	0.1	1.15 (0.97–1.35)	0.12	1.13 (0.96–1.30)	0.12
Female	0.436/0.448	0.64	0.96 (0.79–1.16)	0.75	0.98 (0.82–1.19)	0.58
AF with HT	0.435/0.441	0.77	0.97 (0.81–1.17)	0.80	0.98 (0.83–1.19)	0.70
AF without HT	0.445/0.414	0.14	1.14 (0.96–1.35)	0.12	1.16 (0.96–1.38)	0.11
Lone AF	0.416/0.424	0.72	0.97 (0.81–1.16)	0.70	0.95 (0.79–1.14)	0.66
Other AF	0.455/0.424	0.13	1.16 (0.98–1.37)	0.14	1.14 (0.96–1.35)	0.11

HT: hypertension; 95% CI, 95% confidence interval.

* Uncorrected *P* value and odds ratio (OR).

^†^ Adjusted *P* value with sex, age, hypertension and diabetes by multivariate logistic regression analysis.

^‡^ Empirical *P* value by performing 100,000 Monte-Carlo simulations.

### Genotypic association of SNPs in *TBX5* with AF

The association of the SNPs in TBX5 with AF was further analyzed in three models of inheritance: the dominant, and recessive and additive models. As shown in Table 3, rs7312625 had a significant association with AF in both recessive and additive models. In the recessive model, the *P* value without adjustment was 0.001, and the OR was 1.80. Adjusting for potential confounding factors, the *P* value was 0.01, and the OR was 1.67. In the additive model, the *P*-obs was 0.003, and the *P*-adj was 0.005. The SNP rs880379 had no significant associations with AF any of the three models of inheritance.

**Table 3:** Association of SNPs in *TBX5* with AF with Different Genetic Models of Inheritance.

		Without adjustment*		With adjustment^†^		Empirical *P*^‡^
	Model	*P-obs*	OR (95% CI)	*P-adj*	OR (95% CI)	*P-emp*
rs3825214						
	Dominant	0.188	0.85 (0.68–1.08)	0.107	0.81 (0.63–1.05)	0.110
	Recessive	0.630	0.94 (0.72–1.22)	0.280	0.85 (0.64–1.14)	0.283
	Additive	0.254	0.92 (0.79–1.07)	0.093	0.87 (0.73–1.02)	0.094
rs7312625						
	Dominant	0.61	0.95 (0.80–1.14)	0.25	1.13 (0.93–1.34)	0.63
	Recessive	0.001	1.80 (1.13–2.63)	0.01	1.67 (1.12–2.5)	0.001
	Additive	0.003	n.a.	0.005	1.63 (1.08–2.24)	0.003
rs883079						
	Dominant	0.83	1.03 (0.82–1.82)	0.91	1.02 (0.80–1.28)	0.86
	Recessive	0.10	1.16 (0.97–1.41)	0.06	1.20 (0.99–1.46)	0.11
	Additive	0.19	n.a.	0.24	1.08 (0.95–1.23)	0.20

n.a.: not applicable; 95% CI, 95% confidence interval.

* Uncorrected *P* value and odds ratio (OR).

^†^ Adjusted *P* value with sex, age, hypertension and diabetes by multivariate logistic regression analysis.

^‡^ Empirical *P* value by performing 100,000 Monte-Carlo simulations.

### Snp-snp interaction between rs3825214 and rs7312625 and their association with AF

To study the interaction between rs3825214 (G to A substitution, risk allele = A) and rs7312625 (G to A substitution, risk allele = A), we first defined the association between the number of risk alleles and AF. Then, we used a non-risk allele as a baseline or reference and estimated the OR for 1, 2, 3, and 4 risk alleles (shown in Table 4). The presence of two risk alleles corresponded to a dramatically increased risk for AF (OR = 1.51, P = 0.0047) before and after adjustment for covariates of age, gender, sex, hypertension and diabetes. The highest OR of 2.21 (P<0.0001) was observed for 4 risk alleles. The P value of the trend test was <0.0001.

**Table 4:** Analysis of snp-snp Interactions between rs3825214 and rs7312625.

Number of risk alleles	Control	Case	Sample size	Crude association results					Adjusted association results				
	(*N* = 733)	(*N* = 1265)	(*N* = 1998)	OR	95% CI		z	P	OR	95% CI		z	P^†^
0	137	165	302	Ref.					Ref.				
1	148	183	331	1.03	0.75	1.40	0.16	0.8693	1.02	0.72	1.45	0.13	0.8957
2	201	365	566	1.51	1.13	2.00	2.83	0.0047	1.64	1.19	2.24	3.05	0.0023
3	178	368	546	1.72	1.29	2.29	3.67	0.0002	1.93	1.40	2.65	4.02	0.0001
4	69	184	253	2.21	1.55	3.17	4.36	<0.0001	2.74	1.85	4.07	5.00	<0.0001
Trend test								<0.0001					<0.0001

^†^ *P* value adjusted for sex, age, hypertension and diabetes by multivariate logistic regression analysis.

### Snp-snp interaction among rs3825214, rs7312625 and rs880379 and their association with AF

To study the interactions among rs3825214 (G to A substitution, risk allele = A), rs7312625 (G to A substitution, risk allele = A), and rs883079 (G to A substitution, risk allele = A), we defined the association between the number of risk alleles and AF. We then used a non-risk allele as a baseline or reference and estimated the OR for 1, 2, 3, 4, 5, and 6 risk alleles (shown in Table 5). The presence of 3 risk alleles corresponded to a dramatically increased risk for AF (OR = 1.43, P = 0.0435) before and after adjustment for covariates of age, gender, sex, hypertension and diabetes. The highest OR of 2.35 (P<0.0001) was observed for 6 risk alleles. The P value of the trend test was <0.0001.

**Table 5:** Analysis of snp-snp Interactions among rs3825214, rs7312625 and rs8830799 and Their Association with AF.

Number of risk alleles	Control	Case	Sample size	Crude association results					Adjusted association results				
	(*N* = 733)	(*N* = 1265)	(*N* = 1998)	OR	95% CI		z	P	OR	95% CI		z	P^†^
0	128	139	267	Ref.					Ref.				
1	100	124	224	1.14	0.80	1.63	0.73	0.4657	1.16	0.79	1.72	0.75	0.4534
2	175	286	461	1.50	1.11	2.04	2.63	0.0086	1.61	1.15	2.27	2.77	0.0056
3	104	161	265	1.43	1.01	2.01	2.02	0.0435	1.54	1.05	2.25	2.21	0.0273
4	152	386	538	2.34	1.72	3.17	5.46	<0.0001	2.64	1.89	3.70	5.65	<0.0001
5	27	49	76	1.67	0.99	2.83	1.91	0.0564	2.08	1.15	3.73	2.44	0.0147
6	47	120	167	2.35	1.55	3.56	4.05	0.0001	2.73	1.73	4.31	4.33	<0.0001
Trend test								<0.0001					<0.0001

^†^ *P* value adjusted for sex, age, hypertension and diabetes by multivariate logistic regression analysis.

## Discussion

There are two main types of AF, familial and sporadic AF, which are caused by interactions of genetic and environmental factors. To date, more than 10 causal genes and 20 susceptibility genes for AF have been reported [15]. However, the genetic causes and underlying mechanisms for AF remain largely unknown. We previously reported a TBX5 variant, rs3825214, associated with AF [13]. In this study, we analyzed the association between rs3825214 and AF in a larger cohort. We found that SNP 3825214 was significantly associated with AF (*P*-obs = 0.002, odds ratio [OR] = 0.82), and lone AF (*P*-obs = 6.77x10^-5^, odds ratio [OR] = 0.71). The association of AF with two other TBX5 variants, rs7312625 and rs883079, was also tested. Rs7312625 was associated with lone AF (*P*-obs = 0.015, odds ratio [OR] = 1.27). Using an inheritance model analysis, SNP rs7312625 was significantly associated with AF in both recessive and additive models. SNP rs7312625 was identified in a meta-analysis of African subjects in a genome-wide study, and it was highly correlated with PR interval [8, 9]. Compared to rs3825214, rs7312625 may play a minor role in AF. Additional studies are needed to determine the biological consequence of SNP rs7312625.

In this study, we demonstrated significant interaction among rs3825214, rs7312625 and rs883079. Four-locus risk alleles showed the highest odds ratio when both rs3825214 and rs7312625 were present (P-obs<0.0001, odds ratio [OR] = 2.21). Six-locus risk alleles showed the highest odds ratio in combined rs3825214, rs7312625 and rs 883079(P-obs<0.0001, odds ratio [OR] = 2.35). Significance was established based on a trend test (P<0.0001). PITX2c and ZFHX3 interaction can generate synergistic effects that markedly increase the disease risk of AF [16]. Molecular studies have shown that PITX2c and ZFHX3 participate in a positive feedback loop. Snp-snp interactions in the same gene may be a new mechanism in AF.

Recently, PR interval variation has been associated with an increased risk of long-term AF and heart block [1]. Recent genome-wide association studies for the PR interval duration have been reported, suggesting its association with SNPs in MEIS1, SCN5A, SCN10A and *TBX5/TBX3* [14, 15, 17, 18]. In these studies, several common variants in the *TBX5* gene are associated with the PR interval duration. *TBX5* deletion could lead to longer PR intervals in mice [14]. Mutation in the *TBX5* gene causes Holt-Oram syndrome (HOS), a heart/hand syndrome clinically characterized by upper limb and cardiac malformations [6]. In HOS, the cardiac defects include atrial and ventricular septal defects and AF [19]. Gain-of-function mutations in *TBX5* play a possible role in the development of AF by stimulating TBX3, which is associated with the PR interval and QRS duration [6]. The P1194 p.G125R mutation in *TBX5* increases KCNJ2 expression, potentially contributing to AF [6]. TBX5 can transactivate multiple downstream genes such as ANP and CX40 alone or in synergy with transcriptionally cooperative partners, including NKX2-5 and GATA4 [20–22], and loss-of-function mutations in some target genes and cooperative partners, including ANF, CX40, NKX2-5 and GATA4, have been associated with AF [16]. This result suggests that TBX5 haploinsufficiency is likely an alternative pathological mechanism of AF in a minority of patients. However, the precise molecular mechanisms for TBX5 variants in AF need to be further investigated and established.

For the first time, we report a strong association of SNP rs3825214in the *TBX5* gene with AF and lone AF in a Chinese Han population. Rs7312625 was significantly associated with lone AF. Snp-snp interactions increased the risk of atrial fibrillation. Our data might provide new insights into AF pathogenesis and the design of novel genetic therapies for AF patients.
